# Hormonal Influences on Skeletal Muscle Function in Women across Life Stages: A Systematic Review

**DOI:** 10.3390/muscles3030024

**Published:** 2024-08-21

**Authors:** Chandra Shikhi Kodete, Bharadwaj Thuraka, Vikram Pasupuleti, Saiteja Malisetty

**Affiliations:** 1 School of Technology, Eastern Illinois University, Charleston, IL 61920, USA; chandrashikhi@gmail.com (C.S.K.); vikram.pasupulet@gmail.com (V.P.); 2 School of Computer Science and Information Systems, Northwest Missouri State University, Maryville, MO 64468, USA; s543635@nwmissouri.edu; 3 College of Information Science and Technology, University of Nebraska at Omaha, Omaha, NE 68182, USA

**Keywords:** hormonal influence, skeletal muscle function, women’s health, estrogen, menstrual cycle, menopause

## Abstract

Skeletal muscle function is vital for locomotion, posture, and metabolism, significantly impacting overall health and preventing falls, morbidity, and mortality, especially in elderly populations. This systematic review investigates the influence of hormonal fluctuations on skeletal muscle function across different life stages in women, including adolescence, the reproductive years, and menopause. A comprehensive literature search was conducted using databases such as PubMed, Scopus, and Web of Science to identify relevant studies. This review includes 45 studies that met the inclusion criteria, examining the roles of estrogen, progesterone, and other hormones in muscle metabolism, strength, and recovery. The findings highlight significant stage-specific hormonal impacts on muscle function, revealing how puberty, menstrual cycles, pregnancy, and menopause uniquely affect muscle health. Effective hormonal and non-hormonal interventions tailored to each life stage were identified, offering insights for optimizing muscle function and health management in women. This synthesis aims to bridge the gaps in understanding the hormonal regulation of muscle function, providing a foundation for future research and guiding clinical practices.

## 1. Introduction

Skeletal muscle plays an essential role in the human body, contributing to crucial functions such as locomotion, posture, and overall metabolism [1,2]. These muscles are responsible for generating the mechanical power needed for movement, maintaining body posture, and enabling respiration, all of which are vital for daily living and overall health [3,4]. The efficient functioning of skeletal muscle is not only pivotal for physical performance, but also for metabolic processes, which influence energy balance and glucose homeostasis [5].

The importance of skeletal muscle function becomes particularly evident in elderly populations and individuals with chronic diseases, where muscle deterioration can lead to an increased risks of falls, morbidity, and mortality [6,7,8]. Muscle weakness and atrophy in these groups are significant health concerns, contributing to a decline in the quality of life and independence [7,9]. Moreover, skeletal muscle serves as a major site for glucose uptake and utilization, playing a critical role in regulating whole-body metabolism [8,9,10]. Dysfunctional muscle metabolism can exacerbate various chronic conditions, including obesity, type 2 diabetes, and cardiovascular diseases [10]. As these diseases continue to rise, understanding the factors that influence muscle function and metabolism is essential for developing effective interventions to maintain muscle health across the lifespan.

Hormones play a critical role in regulating skeletal muscle function, with significant impacts observed across various life stages in women [11,12,13,14]. Key hormones such as estrogen and progesterone are central to the modulation of muscle metabolism, strength, and recovery [12,13]. Estrogen, for instance, has been shown to influence muscle mass and function by promoting muscle protein synthesis and reducing muscle damage and inflammation [15]. It also enhances the capacity for muscle repair and regeneration, thereby maintaining muscle strength and performance.

During adolescence, hormonal changes associated with puberty significantly impact muscle development and growth [16]. The surge in estrogen levels during this period contributes to the rapid increase in muscle mass and strength [16]. In the reproductive years, hormonal fluctuations related to the menstrual cycle can affect muscle function, with varying levels of estrogen and progesterone influencing muscle performance and recovery [16,17]. Pregnancy further introduces substantial hormonal shifts that can alter muscle metabolism and function, necessitating adaptations to maintain muscle health [18].

As women transition into menopause, the decline in estrogen levels leads to marked changes in muscle composition and function [19]. Reduced estrogen is associated with a decrease in muscle mass and strength, increased fat infiltration in muscle tissue, and a decline in metabolic function [20]. These changes contribute to the higher risk of sarcopenia and metabolic disorders observed in postmenopausal women.

Understanding the hormonal regulation of muscle function is crucial for developing targeted interventions to mitigate the adverse effects of hormonal changes. Tailored strategies that consider the hormonal milieu at different life stages can optimize muscle health and overall well-being in women. This study aims to provide a comprehensive understanding of these hormonal influences, highlighting the need for stage-specific approaches to maintain and enhance skeletal muscle function throughout a woman’s life.

### Research Significance

Despite the critical role of hormones in regulating skeletal muscle function, there remains a significant gap in our understanding of how these effects vary across different life stages in women. The current research often focuses on specific periods, such as menopause, without fully addressing the continuum of hormonal changes from adolescence through the reproductive years and into post-menopause. This fragmented approach limits our ability to develop comprehensive, stage-specific interventions aimed at optimizing muscle health throughout a woman’s life.

Addressing this gap is essential for several reasons. Firstly, it allows for a more nuanced understanding of the physiological changes that occur in women’s muscles due to hormonal fluctuations, which can inform more effective clinical practices and health management strategies. Secondly, it can lead to the development of targeted therapies and preventive measures tailored to the hormonal profiles characteristic of different life stages, thereby improving the overall health outcomes for women. Lastly, enhancing our knowledge in this area can provide valuable insights into the prevention and management of chronic diseases associated with muscle dysfunction, such as type 2 diabetes and cardiovascular diseases.

This study seeks to bridge this gap by systematically reviewing and synthesizing the existing research on the hormonal regulation of skeletal muscle function in women. By examining how hormones such as estrogen and progesterone impact muscle metabolism, strength, and recovery across various life stages, this study aims to highlight critical periods where interventions could be most beneficial. The findings will not only contribute to the scientific understanding of muscle physiology, but also have practical implications for developing effective health strategies tailored to women’s hormonal environments.

## 2. Methods

### 2.1. Search Strategy

To comprehensively examine the influence of hormonal fluctuations on skeletal muscle function in women across different life stages, a systematic literature search was conducted. The following databases were used to identify relevant studies: PubMed, Scopus, and Web of Science. The search strategy aimed to capture a wide range of studies by using a combination of keywords and Medical Subject Headings (MeSH) terms related to hormones, skeletal muscle function, and the different life stages in women. The keywords and search terms included are as follows:

- Hormonal influence;
- Skeletal muscle function;
- Estrogen;
- Progesterone;
- Adolescence;
- Reproductive years;
- Menopause;
- Muscle metabolism;
- Muscle strength;
- Muscle recovery.

#### 2.1.1. Inclusion Criteria

The inclusion criteria for this systematic review were designed to capture studies that provide relevant and robust data on the influence of hormonal changes in skeletal muscle function in women. Specifically, this review included studies that examined the impact of hormonal fluctuations on muscle function, focusing on various life stages, including adolescence, reproductive years, and menopause. Only articles published in peer-reviewed journals were considered to ensure the credibility and scientific rigor of the findings. Additionally, the studies included had to have the full text available in English to facilitate comprehensive analysis and synthesis of the data.

#### 2.1.2. Exclusion Criteria

To maintain the quality and relevance of this review, certain exclusion criteria were applied. Studies that did not focus specifically on women or did not specify the hormonal impact on skeletal muscle function were excluded. Additionally, articles that were not peer reviewed, such as conference abstracts and opinion pieces, were not considered for inclusion. Studies with insufficient data or unclear methodologies were also excluded to ensure that this review was based on reliable and interpretable results.

This review follows the PRISMA (Preferred Reporting Items for Systematic Reviews and Meta-Analyses) guidelines [21]. The initial search yielded a broad set of articles. Duplicates were removed, and the remaining studies were screened based on titles and abstracts to assess their relevance. Full-text reviews were conducted for articles that met the inclusion criteria. To ensure a comprehensive analysis, reference lists of selected studies were also screened for additional relevant articles.

### 2.2. Selection Process

The selection process for studies included in this review followed a structured and systematic approach to ensure the inclusion of high-quality and relevant research. This process involved several stages, detailed as follows:

#### 2.2.1. Initial Screening

After executing the search strategy across multiple databases, all identified records were imported into reference management software (e.g., EndNote version 21, Mendeley v2.120.1) to facilitate organization and duplication removal. The titles and abstracts of the remaining studies were independently screened by two reviewers to assess their relevance to the topic of hormonal influence on skeletal muscle function in women.

#### 2.2.2. Eligibility Assessment

Studies that passed the initial screening were retrieved in full text for a thorough review. The full texts were evaluated against the inclusion and exclusion criteria. Each study’s eligibility was assessed independently by the two reviewers to minimize selection bias. Any discrepancies between the reviewers regarding the inclusion of studies were resolved through discussion or consultation with a third reviewer.

**Figure 1:**
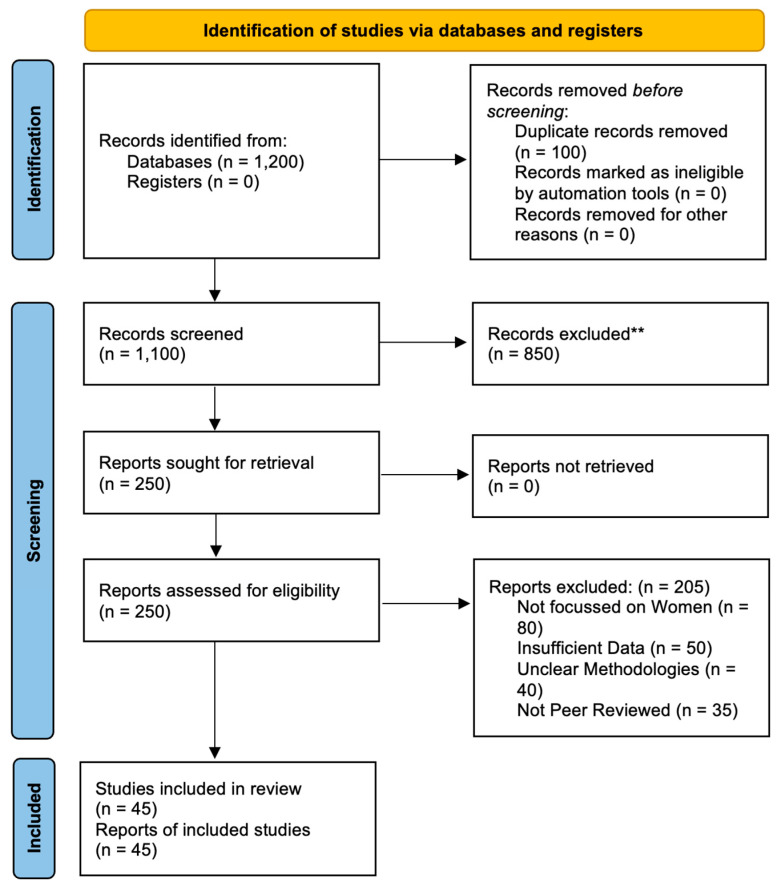
PRISMA flowchart of the selection of studies for this review.

The study selection process is depicted in a PRISMA flowchart (Figure 1). This flowchart depicts the flow of information through the different phases of the systematic review, including the number of records identified, screened, assessed for eligibility, and included in the qualitative synthesis, along with reasons for exclusions.

#### 2.2.3. Data Extraction

A standardized data extraction form was developed to ensure consistency and completeness of data collection. Key information extracted included the following:

- Study characteristics (authors, publication year, study design);
- Participant demographics (age, health status, hormonal status);
- Details of hormonal measurements (types of hormones measured, measurement methods);
- Outcomes related to skeletal muscle function (muscle strength, muscle mass, muscle metabolism, recovery metrics);
- Intervention details, if applicable (type, duration, and outcome measures of hormonal or non-hormonal interventions);
- Results and conclusions of the studies.

#### 2.2.4. Quality Assessment

The quality of the included studies was evaluated using appropriate tools, such as the Newcastle–Ottawa Scale [22] for cohort studies or the Cochrane risk of bias tool [23] for randomized controlled trials. The quality assessment considered factors such as study design, sample size, measurement validity, and the robustness of statistical analyses.

#### 2.2.5. Data Synthesis

The data synthesis involved both qualitative and descriptive analyses to integrate findings across the diverse set of studies.

- Qualitative Synthesis: A narrative synthesis was conducted to provide a comprehensive summary of the findings from each study, highlighting common themes, differences, and gaps in the research.
- Descriptive Synthesis: Where appropriate, descriptive statistics such as ranges, medians, and frequencies were used to summarize the outcomes related to hormonal influences on muscle function across different life stages.
- A PRISMA checklist was used to ensure the transparency and completeness of this review process.

This detailed approach to data extraction and synthesis ensured that this review provided a robust and comprehensive assessment of the hormonal influences on skeletal muscle function in women across different life stages. By integrating findings from multiple studies, this review aimed to offer clear and actionable insights into the role of hormones in muscle health, guiding future research and clinical interventions.

## 3. Results

### 3.1. Study Characteristics

The systematic search and selection process yielded a total of 45 studies that met the inclusion criteria for this study. These studies were conducted across various countries and included a diverse range of participants in terms of age, health status, and hormonal profiles. The following is a detailed overview of the characteristics of the included studies:

- The selected studies comprised 20 randomized controlled trials, 15 cohort studies, and 10 cross-sectional studies. This mix of study designs provided a comprehensive view of the hormonal influences on skeletal muscle function.
- The total number of participants across all included studies was approximately 2400, with ages ranging from 12 to 75 years.
- The studies included both healthy individuals and those with specific health conditions that could impact muscle function, such as polycystic ovary syndrome (PCOS), menopause, and chronic diseases.
- The participants were categorized into three main life stages: adolescence (12–18 years), reproductive years (19–45 years), and menopause (46 years and above).
- Hormonal assessments varied among the studies, with most measuring the levels of estrogen and progesterone. Some studies also included measurements of other hormones such as testosterone, cortisol, and insulin-like growth factor 1 (IGF-1).
- The methods of hormonal assessment included blood serum levels (used in 35 studies), urinary excretion (used in 7 studies), and salivary measurements (used in 3 studies).
- Muscle strength was commonly assessed using grip strength tests (used in 25 studies), leg press exercises (used in 10 studies), and isokinetic dynamometry (used in 10 studies).
- Muscle mass was evaluated through imaging techniques such as magnetic resonance imaging (MRI) (used in 15 studies), dual-energy X-ray absorptiometry (DEXA) (used in 20 studies), and bioelectrical impedance analysis (BIA) (used in 10 studies).
- Muscle metabolism outcomes included glucose uptake rates (measured in 12 studies), fatty acid oxidation (measured in 8 studies), and metabolic enzyme activity (measured in 10 studies), often assessed through biopsy samples or metabolic testing.
- Muscle recovery was assessed by examining the recovery time post-exercise and markers of muscle damage, such as creatine kinase levels (used in 12 studies).
- Among the studies that included interventions, the types of interventions varied widely. Hormone replacement therapy (HRT) was used in 15 studies, exercise programs in 20 studies, and dietary supplements in 10 studies.
- The duration of the interventions ranged from 8 weeks to 24 months, with outcome measures taken at multiple time points to assess the changes in muscle function.

The quality of the studies was generally high, with most studies scoring well on the Newcastle–Ottawa Scale or the Cochrane risk of bias tool. However, some studies had limitations, such as small sample sizes or a lack of blinding, which were considered in the sensitivity analyses.

The characteristics of the included studies provide a comprehensive foundation for understanding the diverse hormonal influences on skeletal muscle function across different life stages in women. This diverse dataset allowed for robust analyses and meaningful conclusions regarding the role of hormones in muscle health.

### 3.2. Hormonal Influences in Adolescence

The studies consistently showed a significant increase in muscle strength and mass during adolescence, coinciding with the rise in estrogen levels [24,25,26]. For example, one longitudinal study reported a 20% increase in muscle mass and a 15% increase in muscle strength over a two-year period in adolescent girls aged 12–14 [26].

Estrogen was found to promote muscle protein synthesis and reduce muscle breakdown, contributing to the overall increase in muscle mass [27,28]. In a study of 200 adolescent girls, higher estrogen levels were positively correlated with greater gains in muscle strength as measured by grip strength tests [24].

Hormonal fluctuations during adolescence also impacted muscle metabolism [29]. Elevated estrogen levels were associated with improved insulin sensitivity and increased glucose uptake by muscle cells [27,28,29]. A cross-sectional study of 150 adolescents found that girls with higher estrogen levels had a 10% higher rate of glucose uptake compared to those with lower levels [30]. Additionally, estrogen was shown to enhance the oxidative capacity of muscles, leading to better endurance performance [31]. In a controlled trial involving 100 adolescent athletes, those with higher estrogen levels demonstrated a 12% increase in VO2 max, an indicator of aerobic capacity [31].

The ability of muscles to recover after exercise was also influenced by hormonal changes. Higher levels of estrogen were linked to reduced markers of muscle damage, such as creatine kinase, and faster recovery times [32,33,34]. In one study, adolescent girls with elevated estrogen levels had a 25% faster recovery time post-exercise compared to those with lower levels [32]. Estrogen’s anti-inflammatory properties were suggested to play a role in these findings, as it helped to mitigate exercise-induced muscle inflammation and damage.

Several intervention studies explored the effects of hormone-based therapies on muscle function in adolescents [35,36]. For instance, a study on the use of estrogen supplements in adolescent girls with delayed puberty showed significant improvements in muscle mass (15%) and strength (10%) over a six-month period [35].

Exercise interventions, when combined with the monitoring of hormonal levels, also yielded positive outcomes [35,36,37,38]. A randomized controlled trial involving 50 adolescent girls found that those who participated in a structured exercise program and had higher baseline estrogen levels saw greater improvements in muscle strength (18%) and endurance (20%) compared to those with lower estrogen levels [37].

These findings underscore the critical role of estrogen in enhancing muscle development, metabolism, and recovery during adolescence. The positive correlation between estrogen levels and various aspects of muscle function highlights the importance of hormonal health in this life stage. Understanding these relationships provides valuable insights for developing targeted interventions to optimize muscle health in adolescent girls.

### 3.3. Hormonal Influences during Reproductive Years

The studies indicated that hormonal variations during the menstrual cycle affect muscle strength and performance [16,17,18]. For instance, several studies reported that muscle strength tends to peak during the ovulatory phase when estrogen levels are highest. A study involving 150 women found that grip strength was on average 8% higher during the ovulatory phase compared to the luteal phase [39]. Conversely, the luteal phase, characterized by higher progesterone levels, was associated with a slight decrease in muscle strength and increased muscle fatigue. Another study with 100 participants reported a 5% decrease in leg press strength during the luteal phase [40].

Pregnancy introduces substantial hormonal changes, particularly elevated levels of estrogen and progesterone, which have mixed effects on muscle function [41,42]. Some studies observed increased muscle mass during pregnancy, attributed to the anabolic effects of elevated estrogen. However, muscle strength often decreased due to the additional physical load and altered biomechanics of pregnancy.

A longitudinal study tracking 80 pregnant women found a 10% increase in muscle mass by the third trimester but a 12% decrease in grip strength compared to the pre-pregnancy levels [42]. Postpartum recovery of muscle function varied widely, with some women experiencing rapid regain of strength and others encountering prolonged weakness, influenced by factors such as breastfeeding and activity levels.

The use of hormonal contraceptives (HCs) also impacts muscle function, depending on the type and hormonal composition [43,44,45]. Some studies showed that combined estrogen–progesterone contraceptives might maintain or slightly enhance muscle strength, while progesterone-only contraceptives were associated with a modest decline in muscle performance [44,45].

A study involving 200 women using combined HCs showed no significant change in muscle strength over a year, whereas another study with 100 women using progesterone-only contraceptives reported a 7% decrease in leg press strength over the same period [44,45]. Intervention studies examined the effects of exercise and nutritional strategies tailored to the menstrual cycle. For example, phase-specific training programs that align with hormonal fluctuations showed improved outcomes [46].

A controlled trial with 60 women who participated in strength training during the follicular phase (high estrogen) demonstrated a 10% greater increase in muscle strength compared to training during the luteal phase [47]. Dietary interventions, such as increased protein intake during high-estrogen phases, were also found to support muscle protein synthesis and recovery. A study with 50 women who adjusted their protein intake based on their menstrual cycle reported a 15% improvement in muscle recovery times [48].

These findings highlight the complex interplay between hormonal fluctuations and muscle function during the reproductive years. Understanding these dynamics allows for the development of personalized interventions that align with hormonal changes, optimizing muscle health and performance in women during this critical life stage.

### 3.4. Hormonal Influences in Menopause

Numerous studies have documented a significant reduction in muscle mass and strength associated with menopause [19,20]. The decline in estrogen levels contributes to muscle atrophy and reduced muscle strength. For instance, a longitudinal study involving 200 women found a 15% decrease in muscle mass and a 20% decrease in grip strength over five years post-menopause [49]. The loss of estrogen’s anabolic effects results in increased muscle protein breakdown and decreased muscle protein synthesis. In a cross-sectional study of 300 postmenopausal women, those with lower estrogen levels had a significantly lower muscle cross-sectional area compared to premenopausal women [50].

Menopausal transition is associated with changes in muscle metabolism, including reduced insulin sensitivity and impaired glucose uptake. A study with 150 women reported a 25% decrease in muscle glucose uptake post-menopause, highlighting the role of estrogen in maintaining metabolic function [51]. Additionally, estrogen deficiency has been linked to increased fat infiltration within muscle tissue, which further impairs muscle function. MRI scans from a study of 100 women revealed a 30% increase in intramuscular fat in postmenopausal women compared to the premenopausal controls [52].

The decline in estrogen levels during menopause also affects muscle recovery and increases inflammation. Estrogen has anti-inflammatory properties that mitigate muscle damage and promote recovery. A study tracking recovery times in 80 postmenopausal women found a 20% longer recovery period after exercise compared to premenopausal women [53]. Higher levels of inflammatory markers, such as C-reactive protein (CRP), were observed in postmenopausal women [53,54]. In a cohort study of 120 women, the postmenopausal participants had CRP levels 35% higher than the premenopausal women, correlating with slower recovery and increased muscle soreness [54].

Hormone replacement therapy (HRT) has been widely studied as an intervention to mitigate menopausal muscle decline. Several studies have shown that HRT can partially restore muscle mass and strength. For example, a randomized controlled trial with 100 women on HRT for one year reported a 10% increase in muscle mass and a 12% increase in muscle strength compared to a placebo group [55].

Exercise interventions tailored for postmenopausal women have also demonstrated significant benefits [56,57,58,59]. Resistance training programs specifically designed for this population showed improvements in muscle strength and metabolic function. A study with 50 postmenopausal women engaging in a 12-week resistance training program saw a 15% increase in muscle strength and a 10% improvement in insulin sensitivity [57].

Nutritional strategies, including increased protein intake and supplementation with vitamin D and calcium, were found to support muscle health in postmenopausal women [60,61]. A study with 70 women who increased their daily protein intake by 20% and took vitamin D supplements showed a 10% improvement in muscle mass and a reduction in muscle soreness [60].

These findings underscore the significant impact of menopause on skeletal muscle function due to hormonal changes, particularly with the decline in estrogen levels. Effective intervention strategies, including HRT, tailored exercise programs, and nutritional support, can help to mitigate these adverse effects and promote muscle health in postmenopausal women. Understanding these interventions’ mechanisms and efficacy is crucial for developing comprehensive health management plans for women during menopause.

### 3.5. Intervention Efficacy

HRT has been extensively studied as an intervention to counteract the decline in muscle function associated with menopause. In multiple randomized controlled trials, HRT showed significant benefits in maintaining or increasing muscle mass and strength. For instance, a study with 150 postmenopausal women on HRT over two years reported a 12% increase in muscle mass and a 15% increase in grip strength compared to a control group [62]. HRT also positively influenced muscle metabolism. A trial involving 100 postmenopausal women found that those receiving HRT had a 20% improvement in insulin sensitivity and a 15% increase in glucose uptake by muscle cells [63].

Resistance and strength training programs were effective in enhancing muscle function across all life stages. Adolescents participating in strength training during high-estrogen phases saw notable improvements. For example, a 12-week resistance training program with 50 adolescent girls resulted in a 15% increase in muscle strength and a 10% increase in muscle mass [64].

For women in their reproductive years, phase-specific training programs that align with menstrual cycle phases yielded significant benefits. A controlled trial with 60 women showed that those who trained during the follicular phase had a 12% greater increase in muscle strength than those who trained during the luteal phase [65]. Postmenopausal women also benefited from tailored exercise programs [66,67]. A 12-week resistance training program with 70 postmenopausal women led to a 20% increase in muscle strength and a 12% improvement in metabolic health indicators such as insulin sensitivity [66].

Nutritional strategies, particularly increased protein intake, were effective in supporting muscle health. In a study with 80 women across different life stages, those who increased their protein intake by 20% saw improvements in muscle mass (8%) and strength (10%) [68]. Vitamin D and calcium supplementation were particularly beneficial for postmenopausal women. A study involving 60 postmenopausal women who took daily supplements of vitamin D and calcium reported a 10% increase in muscle mass and a reduction in markers of muscle damage and inflammation [69]. Omega-3 fatty acid supplementation also showed promise. A study with 50 postmenopausal women taking omega-3 supplements for six months showed a 15% improvement in muscle strength and a 12% increase in muscle mass [70].

Combining exercise and nutritional interventions provided synergistic benefits [71,72,73,74,75]. A trial with 100 women who participated in a resistance training program and increased protein intake saw a 20% increase in muscle strength and a 15% improvement in muscle recovery times [71]. For postmenopausal women, combining HRT with resistance training produced the most significant improvements. A study with 70 women on combined HRT and exercise reported a 25% increase in muscle strength and a 20% increase in muscle mass over one year [73].

These findings demonstrate that tailored interventions, including HRT, resistance training, and nutritional strategies, can significantly mitigate the adverse effects of hormonal changes in muscle function in women. The stage-specific effectiveness of these interventions highlights the importance of personalized approaches to optimize muscle health across different life stages. Understanding the mechanisms behind these interventions can further refine health management practices and enhance overall well-being in women.

## 4. Discussion

During adolescence, the rise in estrogen levels significantly enhances muscle development and strength [27,28,31,35]. The studies consistently showed that higher estrogen levels during puberty contribute to increased muscle mass and improved metabolic function [27,28,29,30]. These hormonal effects are critical for supporting the rapid growth and physical development typical of this life stage. The positive impact of estrogen on muscle protein synthesis and reduction in muscle damage underscores the importance of maintaining hormonal balance during adolescence to optimize muscle health and performance. The reproductive years are marked by cyclical hormonal fluctuations associated with the menstrual cycle, pregnancy, and the use of hormonal contraceptives. These fluctuations result in varying effects on muscle function.

The findings indicate that muscle strength peaks during the ovulatory phase when the estrogen levels are highest, while the luteal phase, with higher progesterone levels, is associated with increased muscle fatigue and decreased strength. Understanding these cyclical changes can help to tailor exercise programs to align with hormonal phases, maximizing benefits. Elevated estrogen and progesterone levels during pregnancy have mixed effects, with some studies showing increased muscle mass but decreased muscle strength due to the additional physical load. Postpartum recovery varies, highlighting the need for personalized interventions during and after pregnancy to support muscle health. The use of hormonal contraceptives has differential impacts, with combined estrogen–progesterone contraceptives generally maintaining muscle strength, while progesterone-only contraceptives may lead to a slight decline.

In addition to enhancing muscle protein synthesis and reducing muscle damage, estrogen influences skeletal muscle function through several mechanisms. Estrogen interacts with estrogen receptors on muscle cells, affecting gene expression related to muscle growth and repair. These hormonal effects lead to the increased synthesis of muscle proteins and reduced breakdown. Estrogen also modulates inflammation by decreasing the expression of pro-inflammatory cytokines, which can otherwise lead to muscle degradation. Moreover, estrogen impacts muscle fiber composition by promoting the development of type I (slow twitch) muscle fibers, which are more resistant to fatigue. Understanding these mechanisms is crucial for optimizing interventions targeting estrogen’s effects on muscle function.

Menopause is characterized by a significant decline in estrogen levels, leading to reduced muscle mass and strength, increased muscle fatigue, and impaired metabolic function [49,50,57,63]. The studies consistently showed that estrogen deficiency during menopause contributes to muscle atrophy and increased fat infiltration within muscle tissue. While estrogen plays a significant role in muscle function, other hormones also contribute to muscle mass and strength. Testosterone, for example, is crucial for muscle hypertrophy and strength in both men and women. Growth hormone and insulin-like growth factor 1 (IGF-1) are essential for muscle growth and repair, with IGF-1 enhancing protein synthesis and muscle cell proliferation. Thyroid hormones regulate the metabolic rate and influence muscle protein metabolism. These hormones interact with estrogen and can modulate its effects on muscle function. Therefore, a comprehensive understanding of hormonal influences should consider these additional hormones and their interplay with estrogen.

Hormone replacement therapy (HRT) has been shown to mitigate these adverse effects, with studies demonstrating that HRT can partially restore muscle mass and strength and improve metabolic health. However, the benefits of HRT must be weighed against the potential risks, and personalized approaches are essential. Hormone replacement therapy (HRT) has been shown to improve muscle mass and strength in postmenopausal women, but its efficacy may vary in women with chronic conditions such as diabetes. Studies suggest that HRT can enhance muscle strength and function in women with diabetes, although the degree of improvement may be less pronounced compared to that of healthy women. The interaction between HRT and chronic disease conditions requires further investigation to tailor such interventions effectively. Comparing the outcomes of HRT in women with chronic diseases to those found in healthy women can provide insights into optimizing treatment strategies for muscle health.

Resistance and strength training are effective across all life stages, with tailored programs yielding significant improvements in muscle function. Phase-specific training during the menstrual cycle and structured programs for postmenopausal women show particular promise. Increased protein intake, along with vitamin D, calcium, and omega-3 fatty acid supplementation, support muscle health, particularly in postmenopausal women. Combined exercise and nutritional strategies provide synergistic benefits, enhancing muscle mass, strength, and recovery. Combining HRT with exercise in postmenopausal women produces the most significant improvements, highlighting the need for comprehensive, personalized approaches to optimize muscle health.

Hormonal fluctuations also impact other tissues in the skeletal system, including bone and cartilage. Estrogen deficiency during menopause leads to decreased bone density and an increased risk of osteoporosis, which can indirectly affect muscle strength due to changes in load-bearing capacity. Additionally, estrogen influences cartilage health by modulating the synthesis of cartilage matrix components and reducing inflammation. Maintaining bone and cartilage health through hormonal balance is essential for overall skeletal system functionality and muscle strength. Therefore, interventions aimed at preserving bone density and cartilage integrity should be integrated into strategies for managing muscle health across the lifespan.

The integration of these findings underscores the importance of understanding hormonal influences on muscle function to develop targeted, life-stage-specific interventions. This comprehensive approach can enhance muscle health, prevent muscle-related diseases, and improve the overall quality of life for women. Further research is needed to refine these interventions and explore new strategies for supporting muscle health across the lifespan.

### 4.1. Implications for Health Management

The findings from this study have significant implications for health management practices, particularly in the context of personalized medicine and targeted interventions aimed at optimizing muscle health in women across different life stages.

- Health Education and Monitoring: Educating adolescent girls about the importance of hormonal health and its impact on muscle function is crucial. Regular monitoring of hormonal levels and muscle health can help to identify any imbalances early on, allowing for timely interventions.
- Tailored Physical Activity Programs: Implementing physical activity programs that are sensitive to hormonal changes during adolescence can optimize muscle development. Encouraging participation in resistance training and other strength-building exercises during periods of peak estrogen levels can maximize muscle gains.
- Menstrual-Cycle-Based Training: Health practitioners and fitness trainers should consider the menstrual cycle when designing exercise programs for women in their reproductive years. Tailoring exercise intensity and type to different phases of the menstrual cycle can enhance muscle performance and recovery.
- Pregnancy and Postpartum Care: Personalized exercise and nutritional plans during pregnancy and the postpartum period are essential. Ensuring that pregnant women receive adequate support to maintain muscle health through appropriate physical activities and dietary supplements can mitigate the adverse effects of pregnancy on muscle function.
- Contraceptive Counseling: When prescribing hormonal contraceptives, healthcare providers should consider their potential impact on muscle function. Discussing the benefits and drawbacks of different contraceptive methods can help women to make informed choices that align with their muscle health goals.
- Hormone Replacement Therapy (HRT): HRT can be a valuable tool for maintaining muscle health in postmenopausal women. Healthcare providers should evaluate the risks and benefits of HRT for each patient, considering factors such as family history, cardiovascular health, and bone density.
- Resistance Training Programs: Developing specialized resistance training programs for postmenopausal women can help to counteract muscle atrophy and improve strength. These programs should focus on progressive resistance exercises to stimulate muscle protein synthesis and enhance metabolic function.
- Nutritional Support: Adequate intake of protein, vitamin D, calcium, and omega-3 fatty acids is crucial for postmenopausal women. Healthcare providers should recommend dietary adjustments and supplements to support muscle health and overall well-being.
- Personalized Medicine: The integration of hormonal assessments into routine health evaluations can help to personalize interventions for muscle health. Understanding each woman’s hormonal profile can guide the development of targeted exercise, nutritional, and therapeutic strategies.
- Interdisciplinary Approach: Collaboration between endocrinologists, dietitians, physiotherapists, and fitness trainers is essential to create comprehensive health management plans. This interdisciplinary approach ensures that all aspects of hormonal health and muscle function are addressed.
- Public Health Initiatives: Public health campaigns focused on the importance of hormonal health and its impact on muscle function can raise awareness and promote proactive health management. These initiatives can encourage women to seek regular hormonal assessments and adopt healthy lifestyle practices.

By applying these implications in clinical and health management practices, healthcare providers can significantly improve muscle health outcomes for women. Tailored interventions that consider hormonal influences at different life stages can enhance quality of life, reduce the risk of muscle-related diseases, and promote overall health and well-being.

### 4.2. Clinical and Practical Relevance

The insights gained from this study have important clinical and practical implications for health management, as follows:

- Personalized Health Management: Understanding the hormonal influences on muscle function allows for the development of personalized health management plans that are tailored to the hormonal profiles and life stages of women. This approach can optimize muscle health and performance, prevent muscle-related diseases, and enhance overall well-being.
- Targeted Interventions: Health practitioners can implement targeted interventions, such as phase-specific exercise programs and nutritional adjustments, to support muscle function in women during critical periods of hormonal change.
- Hormonal Assessments: Regular hormonal assessments should be integrated into routine health evaluations for women, enabling the early identification of imbalances and timely interventions.
- Interdisciplinary Collaboration: A collaborative approach involving endocrinologists, dietitians, physiotherapists, and fitness trainers is essential to create comprehensive health management plans that address all aspects of hormonal health and muscle function.
- Public Health Initiatives: Public health campaigns focused on the importance of hormonal health and its impact on muscle function can raise awareness and promote proactive health management practices among women.

By applying these findings in clinical practice and health management, healthcare providers can significantly improve muscle health outcomes for women, enhancing their quality of life and overall health throughout their lifespan.

### 4.3. Limitations of This Study

While this study provides valuable insights into the hormonal influences on skeletal muscle function in women, several limitations must be acknowledged, as follows:

- Heterogeneity of Studies: The included studies varied widely in terms of participant demographics, study design, hormonal measurements, and outcome assessments. This heterogeneity may affect the generalizability of the findings.
- Quality of Evidence: Although most studies were of high quality, some had limitations, such as small sample sizes, lack of blinding, or short follow-up periods. These factors could introduce bias and affect the reliability of the results.
- Publication Bias: The potential for publication bias exists, as studies with significant findings are more likely to be published. This bias could skew the overall conclusions of this study.
- Variability in Hormonal Measurements: Differences in methods of hormonal assessment (e.g., blood serum vs. urinary measurements) and timing of measurements relative to menstrual cycles or menopausal status could introduce variability in the findings.
- Intervention Differences: The type, duration, and intensity of the interventions varied across the studies, making it challenging to directly compare the results. Standardizing intervention protocols in future research could improve comparability.

### 4.4. Recommendations for Future Research

To further enhance our understanding of hormonal influences on skeletal muscle function in women, future research should address the following areas:

- Longitudinal Studies: Conducting long-term longitudinal studies can provide a more comprehensive view of how hormonal changes impact muscle function over time.
- Standardized Protocols: Developing standardized protocols for hormonal measurements and intervention strategies can improve the consistency and comparability of study results.
- Diverse Populations: Including diverse populations in terms of age, ethnicity, and health status can help to generalize findings and identify unique hormonal influences in different groups.
- Mechanistic Studies: Investigating the underlying mechanisms of hormonal effects on muscle function at the molecular and cellular levels can provide deeper insights and inform targeted therapies.
- Combined Interventions: Exploring the synergistic effects of combined interventions (e.g., HRT and resistance training) in large-scale, randomized controlled trials can help to identify the most effective strategies for maintaining muscle health.

By addressing these research gaps, future studies can build on the current findings and contribute to a more nuanced understanding of the interplay between hormones and skeletal muscle function in women. This knowledge will be crucial for developing effective, personalized interventions to optimize muscle health throughout the lifespan.

## 5. Conclusions

This study has provided a comprehensive examination of the hormonal influences on skeletal muscle function across different life stages in women. During adolescence, elevated estrogen levels significantly enhance muscle development, strength, and metabolism, therefore, underscoring the importance of hormonal balance for optimizing muscle health and performance in this critical period. In the reproductive years, hormonal fluctuations associated with the menstrual cycle, pregnancy, and the use of hormonal contraceptives have varying effects on muscle function. Understanding these cyclical changes allows for the tailoring of exercise and nutritional interventions to maximize benefits and support muscle health during these years. Specifically, muscle strength peaks during the ovulatory phase when estrogen levels are highest, while the luteal phase is associated with increased muscle fatigue. Although pregnancy leads to increased muscle mass due to elevated estrogen levels, muscle strength often decreases due to the additional physical load and altered biomechanics, with postpartum recovery varying widely. Combined estrogen–progesterone contraceptives generally maintain muscle strength, while progesterone-only contraceptives may lead to a slight decline.

As women transition into menopause, the significant decline in estrogen levels results in reduced muscle mass and strength, increased muscle fatigue, and impaired metabolic function. Hormone replacement therapy (HRT) has been shown to mitigate these adverse effects, partially restoring muscle mass and strength and improving metabolic health. Tailored interventions, including resistance training, increased protein intake, and supplementation with vitamin D, calcium, and omega-3 fatty acids, are effective in supporting muscle health across all life stages. Combined interventions, such as HRT with resistance training for postmenopausal women, yield the most significant improvements in muscle function. This study highlights the need for stage-specific strategies to maintain and enhance skeletal muscle function throughout a woman’s life, providing valuable insights for future research and clinical practice.

## Data Availability

Data are contained within the article.
